# Lack of Amino Acid Alterations Within the Cochlear Nucleus and the Auditory Cortex in Acoustic Trauma-Induced Tinnitus Rats Using In Vivo Microdialysis

**DOI:** 10.3390/audiolres14060083

**Published:** 2024-11-17

**Authors:** Shanshan Yuan, Huey Tieng Tan, Paul F. Smith, Yiwen Zheng

**Affiliations:** 1 Department of Pharmacology and Toxicology, Faculty of Biomedical and Molecular Sciences, University of Otago, Dunedin 9054, New Zealandtanhu311@student.otago.ac.nz (H.T.T.);; 2 Brain Health Research Centre, University of Otago, Dunedin 9054, New Zealand; 3 The Eisdell Moore Centre for Research on Hearing and Balance Disorders, University of Auckland, Auckland 1010, New Zealand

**Keywords:** amino acids, tinnitus, acoustic trauma, cochlear nucleus, auditory cortex, in vivo microdialysis

## Abstract

**Background/Objectives:** Tinnitus is a debilitating auditory disorder commonly described as a ringing in the ears in the absence of an external sound source. Sound trauma is considered a primary cause. Neuronal hyperactivity is one potential mechanism for the genesis of tinnitus and has been identified in the cochlear nucleus (CN) and the auditory cortex (AC), where there may be an imbalance of excitatory and inhibitory neurotransmissions. However, no study has directly correlated tinnitus with the extracellular levels of amino acids in the CN and the AC using microdialysis, which reflects the functions of these neurochemicals. In the present study, rats were exposed to acoustic trauma and then subjected to behavioural confirmation of tinnitus after one month, followed by microdialysis. **Methods:** Rats were divided into sham (aged, *n* = 6; young, *n* = 6); tinnitus-positive (aged, *n* = 7; young, *n* = 7); and tinnitus-negative (aged, *n* = 3; young, *n* = 3) groups. In vivo microdialysis was utilized to collect samples from the CN and the AC, simultaneously, in the same rat. Extracellular levels of amino acids were quantified using high-performance liquid chromatography (HPLC) coupled with an electrochemical detector (ECD). The effects of sound stimulation and age on neurochemical changes associated with tinnitus were also examined. **Results:** There were no significant differences in either the basal levels or the sound stimulation-evoked changes of any of the amino acids examined in the CN and the AC between the sham and tinnitus animals. However, the basal levels of serine and threonine exhibited age-related alterations in the AC, and significant differences in threonine and glycine levels were observed in the responses to 4 kHz and 16 kHz stimuli in the CN. **Conclusions:** These results demonstrate the lack of a direct link between extracellular levels of amino acids in the CN and the AC and tinnitus perception in a rat model of tinnitus.

## 1. Introduction

Subjective tinnitus is the perception of a phantom sound, negatively affecting more than 20% of the world’s population. Multiple risk factors, including head and neck injuries, otologic diseases, and certain medications, can induce tinnitus; however, occupational- or leisure-related sound overexposure is the most common trigger [1]. A variety of treatments are available to help people adapt to and modulate their tinnitus perception, but no proven treatments that reliably eliminate tinnitus itself have been established at present. Additionally, there is no single drug that has been approved by the Food and Drug Administration (FDA) or European Medicines Agency (EMA) on the market targeting tinnitus. Understanding the mechanisms behind tinnitus perception may help to develop strategies for effective treatment.

The mechanisms for the genesis of tinnitus are complex and still not entirely understood, but it is believed to involve neuronal hyperactivity developed at several neural stations along the auditory pathways. Numerous studies have investigated the involvement of the cochlear nucleus (CN), the first auditory nucleus in the central auditory system, in the development of tinnitus, especially in the dorsal cochlear nucleus (DCN) [2]. The earliest study reported that the loudness of tinnitus could be changed by direct electrical stimulation of the DCN [3]. Subsequently, neural hyperactivity has been observed in the DCN in tinnitus animals [4,5,6], and the DCN has also been found to exhibit several forms of neural plasticity that parallel the traits of tinnitus [7], including injury-induced plasticity, temporal plasticity, stimulus-dependent plasticity, and modulatory plasticity. However, the fact that DCN lesions prior to acoustic trauma could prevent tinnitus generation, but failed to reverse established tinnitus in rats [8,9], suggests that although the DCN may be essential in tinnitus generation, it may not be necessary for chronic tinnitus maintenance and perception.

On the other hand, the auditory cortex (AC), which is the last auditory region in the ascending auditory pathways, has been suggested to play a role in the generation and perception of tinnitus. For example, an increased spontaneous firing rate and synchrony as well as tonotopic reorganization have been reported in the AC of noise-induced tinnitus animals [10,11,12,13], and stimulation of the AC suppressed tinnitus in both animals [14] and humans [15]. Moreover, animals with behaviourally confirmed tinnitus were found to exhibit a decreased inhibitory synaptic transmission in the tinnitus frequency region of the AC, and the reduced inhibitory synaptic transmission was correlated with a reduced expression of the GABA-synthesizing enzyme, glutamic acid decarboxylase, in the same region [16]. Furthermore, the systemic administration of a GABA-enhancer drug (an inhibitor of GABA transaminase), vigabatrin, 15 min before the tinnitus behavioural testing, was able to abolish tinnitus perception in such animals [16].

Therefore, neurochemical alterations within the auditory brain regions might be one of the factors underlying tinnitus generation. To date, most of the neurochemical studies in tinnitus research focus on the changes in amino acids, as they are the most abundant neurotransmitters in the central auditory system [17]. For example, glutamate increased in the ventral cochlear nucleus (VCN) at 1 week but decreased at 2 weeks then recovered at 3 months after noise exposure in chinchillas [18], and elevated aspartate, glutamate, and GABA and reduced taurine concentrations were found in the CN and the AC of hamsters at 5 months, following intense sound exposure [19]. In addition, taurine, serine, threonine, and alanine were also found to decrease with a long-term (1–1.5 months), but not a short-term (2 h or 1 day), time point after noise exposure in the CN [20]. However, none of these studies measured amino acids in the extracellular space, even though variations in extracellular levels may correlate with changes in neuronal activity such as neurotransmissions. In a recent study from our laboratory, time-dependent effects of acoustic trauma and tinnitus on extracellular levels of amino acids were investigated in the inferior colliculus (IC) of rats using in vivo microdialysis [21]. Although acoustic trauma caused a significant increase in GABA up to 1 week post-acoustic trauma, there was no significant change in GABA levels at 5 months post-acoustic trauma in tinnitus animals [21]. To the best of our knowledge, the present study is the first time extracellular amino acid levels have been measured in the CN and the AC simultaneously in the same rats using in vivo microdialysis and compared between sham and tinnitus-positive groups.

## 2. Materials and Methods

All procedures were approved by the University of Otago Committee on Ethics in the Care and Use of Laboratory Animals (approval number: AEC#19/2011; approval date: 1 February 2020). All methods were performed in accordance with the relevant guidelines and regulations. We confirm that this study is reported in accordance with the ARRIVE guidelines (https://arriveguidelines.org, accessed on 1 March 2020).

Thirty-two male Wistar rats, aged between 8–10 weeks (300–350 g at the beginning of the experiment), were obtained from the Hercus Taieri Resource Unit, University of Otago, Dunedin, New Zealand. The rats were housed in pairs and maintained in an animal facility with a 12/12 h light/dark schedule under a regulated temperature (21 ± 1 °C) and humidity (55 ± 5%). All animals were given ad libitum access to food and water, except when they had restricted access to water throughout the behavioural test for tinnitus.

**Figure 1:**
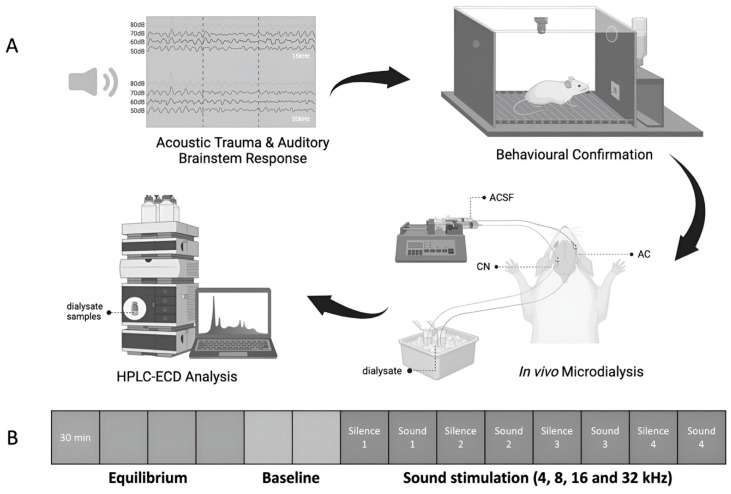
Illustration of (**A**) the experimental design and (**B**) the timeline of dialysis sample collection. One month after exposure to either acoustic trauma or a sham procedure, tinnitus-like behaviour was assessed using conditioned lick suppression. One week later, a third ABR confirmed the recovery of hearing levels. Subsequently, in vivo microdialysis experiments were conducted, collecting dialysate samples from the CN and the AC of animals for HPLC–ECD measurement to quantify amino acid neurochemical levels. Samples were collected during a 2 h equilibration, and then baseline samples were collected every 30 min for 1 h. Following this, 30 min sound stimulations with varied frequencies were delivered to either the ear exposed to acoustic trauma or the sham-exposed ear, in a random order. Acoustic trauma was confined to the ipsilateral ear using an ear tube. A 30 min silence period followed each stimulation to allow for the delivery of the corresponding dialysate to the outlet tube after the initial sample collection.

Due to the quarantine period of COVID-19, the study was conducted on two batches of rats with a 2-month age difference between them, making age a necessary factor to be considered in this study. Therefore, thirty-two rats were randomly allocated into four groups: (1) sham aged, *n* = 6; (2) sham young, *n* = 6; (3) exposed aged, *n* = 10; and (4) exposed young, *n* = 10. The animals received either acoustic trauma or the sham procedure under anaesthesia, and hearing levels were measured before and immediately after the acoustic trauma or sham procedure using acoustic brainstem-evoked responses (ABRs). One month later, they were assessed for the perception of tinnitus using a conditioned lick-suppression paradigm, which took 4–6 weeks to complete. One week after the completion of the tinnitus testing, the ABR was measured again to verify the recovery of hearing levels. Subsequently, in vivo microdialysis experiments were conducted, and the dialysate samples were collected simultaneously from the CN and the AC of the same animal under anaesthesia. The levels of amino acids in the microdialysate were quantified using high performance chromatography (HPLC) coupled with an electrochemical detector (ECD) (Figure 1A).

Unilateral acoustic trauma was delivered using the techniques previously described by Bauer and Brozoski [4] and our previous publications, e.g., [21,22,23]. The rats were anaesthetised with a ketamine (75 mg/kg and 100 mg/mL, s.c.) and dormitor (0.3 mg/kg and 1 mg/mL, s.c.) mixture, prior to the sham procedure or acoustic trauma. A pure 16 kHz tone with an intensity of 115 dB (RZ6 multi-I/O processor, Tucker-Davis Technologies, Alachua, FL, USA) was delivered to one of the ears for 1 h through a closed-field magnetic speaker with a tapered tip (Tucker-Davis Technologies) attached to a 3 mm cone-shaped speculum that fitted tightly into the external auditory canal. Acoustic values were calibrated before acoustic trauma by a pre-polarised free-field microphone (Type 40 BE, GRAS Sound & Vibrations, DK-2840 Holte, Denmark). The unexposed ear was plugged with a foam earplug during the sound exposure. To reduce lateralisation bias, the noise exposure was counterbalanced between the left and right ears. The sham animals received an identical anaesthesia procedure and duration as the acoustic trauma animals, but without noise exposure, in the same sound-attenuated chamber.

Acoustic brainstem-evoked responses (ABRs) were measured in all rats in both ears before and immediately after acoustic trauma. Using an identical anaesthesia technique and setup as described above, three stainless-steel subdermal needle electrodes (14 × 0.38 mm, Technomed) were inserted subcutaneously at the vertex and over the bullae below the recording ear, and the same position below the other ear was used for the reference electrode, and at the occiput, as the ground electrode. Tone bursts of a 5 ms duration (2 ms rise/decay and 1 ms plateau), presented at a rate of 21/s, were employed to examine the ABR thresholds of the animals. These tone bursts were presented at four different frequencies (8, 16, 20, and 32 kHz, respectively) in a series of decreasing intensities, starting with a level that elicited an observable evoked potential. Hearing thresholds were defined as the lowest intensity that produced a visually distinct potential, progressing in 20, 10, and 5 dB steps. At the end of the ABR measurement, the anaesthesia was reversed by an injection of antisedan (1 mg/kg and 5 mg/mL, s.c.).

The presence of tinnitus was assessed by a conditioned lick-suppression paradigm, as described in our previous publications, e.g., [21,22,23]. Briefly, the animals were subjected to water restriction and were allowed to drink in an operant conditioning testing chamber during behavioural testing. The conditioned lick-suppression paradigm consisted of 15 min of testing, and the animals went through 3 phases, including acclimation, conditioned lick-suppression training, and frequency discrimination. A constant 60 dB SPL broad band noise (BBN) was played as a background noise throughout each 15 min session except at 10 intervals, at which point 15 s acoustic stimuli presentations or silence was randomly allocated (Figure 1B). Two of the 10 presentations were always speaker-off periods (i.e., silence), and the remaining 8 were either BBN, 20 kHz tones, or 32 kHz tones at 4 different intensity levels (40, 50, 60, and 70 dB SPL for BBN; 60, 70, 80, and 90 dB SPL for 20 kHz; and 70, 80, 90, and 100 dB SPL for 32 kHz) in a random order, with each stimulus presented twice within each session. The stimulus was not delivered in the first or last minute of the session. The type of stimulus varied between sessions but remained constant within a session. The animals had 3 sessions of acclimation for each type of stimulus before moving to the conditioned lick-suppression training, where the acoustic stimulus was presented in the same way as acclimation, except that a 3 s mild foot shock (0.3–0.45 mA) was presented at the end of each speaker-off (silence) period. The foot shock acted as an unconditioned stimulus, and the animals learned the association between the speaker-off period and the foot shock by suppressing the licking during the silence period. The number of licks during the 15 s preceding the stimulus presentation and during the 15 s stimulus-presentation period were recorded using an electronic photo beam. The lick-suppression ratio was calculated by comparing the number of licks between these two periods:

$$SR=\frac{B}{A+B}$$

A = the number of licks during the preceding period;
B = the number of licks during the stimulus presentation period.

Once a stable lick suppression was established (SR < 0.2), the animals underwent a frequency discrimination phase. The acoustic stimuli presented in the discrimination phase were identical to the previous phases, except that the animal received a mild foot shock only when their SR ratio was 0.1 or above during the speaker-off period. Frequency discrimination curves were composed based on the SRs for each acoustic stimulus presented.

The animals were anaesthetised with urethane (1.5 g/kg, i.p.), and the body temperature of the animals was maintained at 37.0 ± 0.5 °C using a thermostatically regulated heating pad (Harvard Apparatus, Holliston, MA, USA) throughout the procedure. The animal was secured to the stereotaxic frame using hollow ear bars, and xylocaine (0.1 mL, with 1:100,000 adrenaline) was injected, s.c., along the incision line. An incision was made along the midline of the head, and the periosteum was removed to expose the skull. Two small holes were drilled to allow the implantation of a microdialysis guide cannula into the primary AC (ML = 6.8 mm, AP = −4.5 mm, and DV = 4.5 mm) and the CN (ML = 3.8 mm, AP = −11.3 mm, and DV = 7.8 mm), respectively, using the Paxinos Rat Brain Atlas [24]. The microdialysis probes were then inserted into the guide cannula to collect the dialysate samples. Throughout the course of sample collection, 3 mL of saline (0.9% NaCl; Baxter International Inc., Auckland, New Zealand) was applied, s.c., every 4 h in order to prevent dehydration, and 0.5 mL of urethane was supplemented every 4 h to keep the animal under anaesthesia. The toe-pinch reflex, body temperature, and corneal dryness were monitored continually throughout the surgery.

Initially, a 2 h equilibration period established a stable baseline after probe insertion. Subsequently, two baseline dialysate samples were collected every 30 min. Afterwards, sound stimulation was delivered for 30 min at each frequency (4, 8, 16, and 32 kHz; at 75 dB SPL; 5 pulses/s, with a pulse duration = 5 ms and with a 1 ms rise/fall time) in the ear which had been exposed to acoustic trauma or the sham procedure beforehand (Figure 1B). Each sound-stimulation period was separated by a 30 min silence period. Samples were stored in a −80 °C freezer until high-performance liquid chromatography (HPLC) analysis.

The mobile phase contained a 28%-methanol, 2%-acetonitrile, and 100 mM di-sodium hydrogen phosphate anhydrous (Na_2_HPO_4_, VWR Chemicals) buffer in MilliQ water, with the pH adjusted to 6.75 using HPLC-grade orthophosphoric acid (Fisher Scientific, Loughborough, UK). Ten μL of the internal standard (homoserine, 1 ng/µL) was added to the amino acid standards or the samples, followed by a pre-column derivatisation reaction, which was achieved by mixing 40 μL of the o-phthalaldehyde (OPA) (Sigma, Tokyo, Japan) with the standards or samples and incubating for 3 min. Fifty µL of the mixture was injected into the HPLC system, and the derivatised amino acids were detected by the ECD using the following settings: a guard-cell potential of +650 mV with a gain range of 100 nA, an E2 potential of +200 mV with a gain range of 100 μA, and an E3 potential of +700 mV with a gain range of 500 nA. The amino acids were identified based on their retention times and quantified according to the standard curve generated by the Chromeleon 7.3 software.

A statistical analysis was conducted on the ABR thresholds, frequency discrimination curves, and amino acid levels in response to sound stimulation using SPSS 27 (Chicago, IL, USA). The baseline levels of amino acids were analysed using GraphPad Prism v.9.5.1 (Boston, MA, USA). All data were tested for the assumptions of normality using Kolmogorov–Smirnov and Shapiro–Wilk tests. The non-normal data were square root (sqrt) values or log-transformed to meet the criterion for a statistical analysis.

A linear mixed model (LMM) analysis, using a restricted maximum likelihood procedure, was carried out in preference to repeated measure ANOVAs because of the problem caused by extensive autocorrelations in repeated-measure data. LMM analyses model the covariance structure of the repeated-measure data in order to address this problem [25]. Akaike’s Information Criterion (AIC) estimates the goodness of fit of a given model, and the most appropriate covariance matrix structure was used based on the smallest AIC value. Using LMM, the ABR data were analysed with exposure (sham or exposure) and age (aged or young) as the between-group fixed factors, with frequency (8, 16, 20, or 32 kHz); side (ipsilateral or contralateral); and time (pre-acoustic trauma, immediately post-acoustic trauma, or 3 months following acoustic trauma) as the repeated measures. The tinnitus testing data were analysed with group (sham or tinnitus-positive) and age (aged or young) as the between-group fixed factors and intensity (0, 40, 50, 60, and 70 dB SPL for BBN; 0, 60, 70, 80, and 90 dB SPL for 20 kHz; and 0, 70, 80, 90, and 100 dB SPL for 32 kHz) as the repeated measures. Before the analysis of the amino acid data, the Grubbs’ test was conducted to detect and remove the outliers in a univariate data set that followed an approximately normal distribution, with alpha < 0.01. The basal amino acid data were analysed using a two-way ANOVA, with group (sham and tinnitus-positive) and age (aged and young) as the fixed factors. The amino acid data, in response to external sound stimulation, were expressed as a percentage of the corresponding sound-off period preceding each sound-on period and were analysed using an LMM analysis, with group (sham and tinnitus-positive) and age (aged and young) as the between-group fixed factors and frequency (4, 8, 16, and 32 kHz) as the repeated measures. For all data, statistical significance was set as *p* ≤ 0.05.

The animals were euthanized at the end of the in vivo microdialysis experiment, and brains were removed and placed in 10% phosphate-buffered formalin at 4 °C for 3 days, followed by 0.1 M phosphate buffer (PB) containing 30% sucrose at 4 °C until the brain sank. Brains were then embedded in an optimal cutting temperature (OCT) compound and sectioned and stained with cresyl violet. The placement of the microdialysis probes was examined using a Microfiche Reader. Partly due to the disruptions of COVID-19, which entailed isolation for 2 months in New Zealand, only a few samples of the brains were analysed histologically, although this was counterbalanced and randomized across groups (i.e., the brains which were not analysed were Missing at Random (MAR)), which means that the histological analysis was not biased toward any single group [25].

## 3. Results

### 3.1. Hearing Levels Following Acoustic Trauma

**Figure 2:**
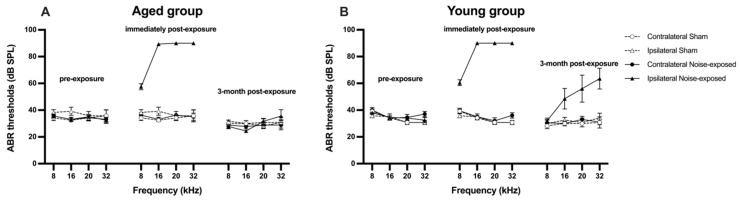
Acoustic brainstem-evoked response (ABR) thresholds for the contralateral and ipsilateral ears of (**A**) aged and (**B**) young rats at three different time points: before, immediately after, and at 3 months after acoustic trauma and sham procedure. Data are presented as means ± SEM.

Immediately following acoustic trauma, there was a significant upward shift in the ABR thresholds in the exposed rats compared to the sham rats, indicated by a significant exposure effect (F_1, 324.642_ = 165.370, *p* ≤ 0.001, see Figure 2). The elevation in the ABR thresholds was specifically related to the ipsilateral ear of the exposed rats, as evidenced by a significant side effect (F_1, 324.642_ = 188.530, *p* ≤ 0.001). The degree of the ABR threshold increase was significantly greater at 16 kHz, 20 kHz, and 32 kHz than at 8 kHz in the ipsilateral ear of noise-exposed rats, with a significant frequency x side effect (F_3, 186.301_ = 10.187, *p* ≤ 0.001) and an exposure x frequency effect (F_3, 186.301_ = 7.413, *p* ≤ 0.001). When the hearing level was assessed again at 3 months after acoustic trauma, it had returned back to normal in the aged rats. By contrast, the hearing level in the ipsilateral ear of the young, exposed rats was still significantly impaired compared to the young sham rats (Figure 2), which was illustrated by a significant age x exposure interaction (F_1, 324.642_ = 15.766, *p* ≤ 0.001).

### 3.2. Behavioural Confirmation of Tinnitus

**Figure 3:**
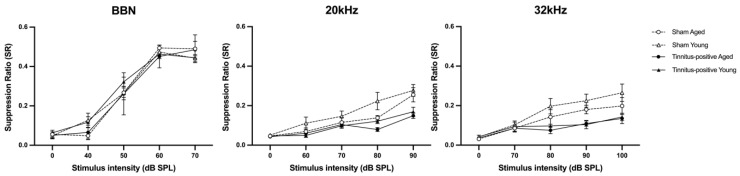
Frequency discrimination curves of animals after the confirmation of tinnitus-like behaviour at one month following sound overexposure or sham procedure, for sham aged, sham young, tinnitus-positive aged, tinnitus-positive young rats, in response to BBN and 20 kHz and 32 kHz tones, respectively. Results are shown as a function of stimulus intensity in dB SPL and frequency in kHz. Data are presented as means ± SEM.

The presence of tinnitus-like behaviour in the rats was assessed through the conditioned lick-suppression paradigm at one month following unilateral acoustic trauma. The frequency discrimination curves were plotted using the SR in relation to different testing-stimulus intensities. Based on the criterion that an animal was deemed to have tinnitus if its SR curve was more than one SD below the mean SR curve of the sham group, 70% of the acoustic trauma-exposed rats (14 out of 20; aged, *n* = 7; young, *n* = 7) exhibited behavioural evidence of tinnitus. The mean discrimination curves were compared between the sham and tinnitus-positive groups for BBN, 20 kHz, and 32 kHz stimuli for the aged and young rats, accordingly. The discrimination curves of the tinnitus groups were significantly downward shifted in the aged and young rats, respectively (Figure 3), which was evident by a significant group effect at 20 kHz (F_2, 26.000_ = 5.804, *p* ≤ 0.008) and 32 kHz (F_2, 27.633_ = 5.700, *p* ≤ 0.008). This suggests that the rats in the tinnitus-positive group experienced tinnitus at high frequencies. Moreover, the young rats showed greater suppression of the discrimination curve at 20 kHz, as indicated by a significant age effect for 20 kHz (F_1, 26.000_ = 8.121, *p* ≤ 0.008).

### 3.3. Extracellular Amino Acid Levels in the CN and the AC of Tinnitus Rats

**Figure 4:**
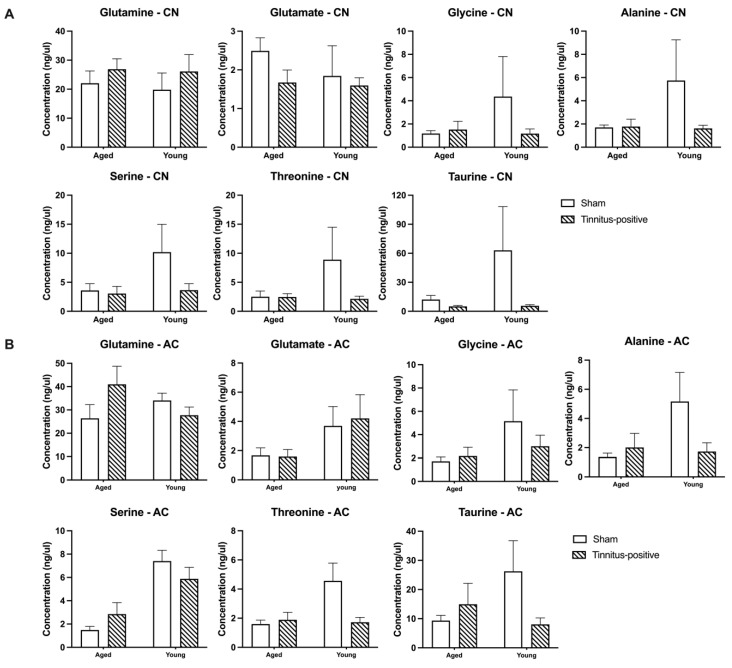
Extracellular basal concentrations for glutamate, serine, glutamine, glycine, threonine, taurine, and alanine in (**A**) the CN and (**B**) the AC of sham and tinnitus-positive rats of different ages. Data are presented as means ± SEM.

Three months following acoustic trauma, the basal extracellular concentrations of seven amino acids were compared within the CN and the AC between the sham and tinnitus-positive rats of the two different age groups. There were no significant differences in any of the amino acids examined between the sham and tinnitus-positive rats, indicating that the presence of tinnitus had no effect on the basal amino acid extracellular levels in the CN and AC of either aged or young rats (Figure 4A,B). However, serine levels were lower in aged animals in the AC in both sham and tinnitus-positive animals (significant age effect, F_1, 14_ = 19.44, *p* = 0.0006), while threonine levels were lower in the aged animals in the sham group only not in the tinnitus-positive group (significant age effect, F_1, 19_ = 4.886, *p* = 0.0395; significant group and age interaction, F_1, 19_ = 6.1, *p* = 0.0223).

**Figure 5:**
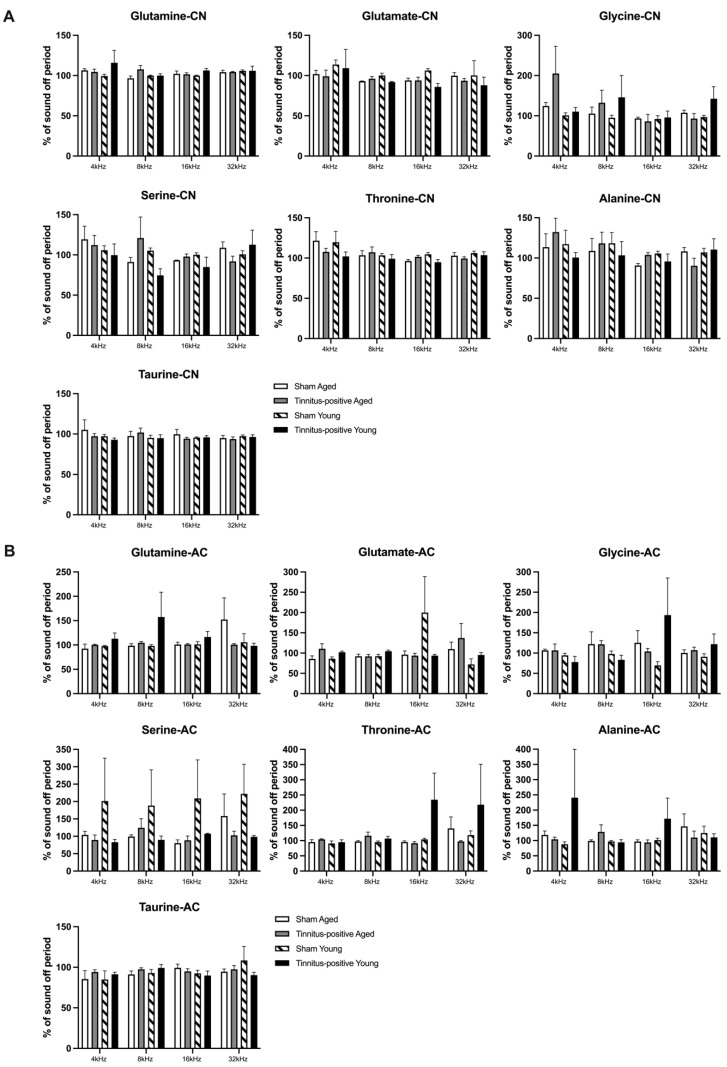
Effects of sound stimulations on the extracellular levels of glutamate, serine, glutamine, glycine, threonine, taurine, and alanine in (**A**) the CN and (**B**) the AC of sham and tinnitus-positive rats of different ages. Data are shown as a percentage of the baseline concentration, presented as means ± SEM.

When the animals were given sound stimulations of different frequencies, threonine levels varied significantly with the frequency shift in the CN (Figure 5A), as indicated by a significant frequency effect (F_3, 37.998_ = 3.435, *p* ≤ 0.026). Pairwise comparisons revealed that when the rats were exposed to sound stimuli, the threonine concentration was significantly higher in response to 4 kHz than to 16 kHz (*p* ≤ 0.007). In addition, the sound stimuli also significantly altered the level of glycine, with higher concentrations observed in response to 4 kHz than to 16 kHz, as evident by a pairwise comparison (*p* ≤ 0.019). However, neither tinnitus nor age altered the amino acid concentrations in the CN in response to sound stimulation (Figure 5A). In the AC, the levels of the seven amino acids measured did not exhibit any significant tinnitus- or age-related changes following sound stimulation (Figure 5B).

### 3.4. Histological Analysis

**Figure 6:**
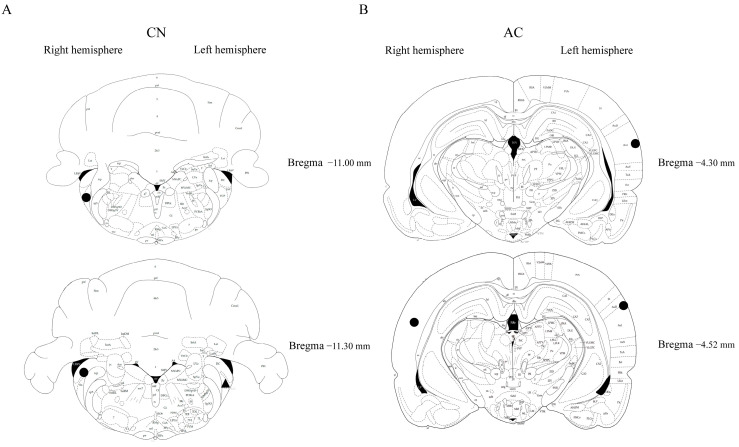
Placement confirmation of microdialysis probes in (**A**) the CN and (**B**) the AC. Symbols: circle (for tinnitus-positive) and triangle (for sham).

Cresyl violet staining was carried out to verify the positioning of the microdialysis probes within the CN and AC. Due to the time constraints caused by the COVID-19 lockdown, limited data were collected; however, they were collected in a random manner. The successful placement of the microdialysis probes in the CN and AC regions was confirmed (Figure 6), including three tinnitus rats in the AC (tinnitus-positive aged, *n* = 2; tinnitus-positive young *n* = 1) and two tinnitus rats and one sham rat in the CN (tinnitus-positive aged, *n* = 2; sham aged *n* = 1). Due to the consistent use of the same stereotaxic coordinates, we are confident that all of the microdialysis probes were placed accurately.

## 4. Discussion

A pure tone of 16 kHz at 110 dB SPL was delivered to rats unilaterally for 1 h in the present study, which caused an acute hearing loss in the exposed ear of acoustic trauma-exposed rats across all the frequencies tested. This observation of hearing loss after acoustic trauma was generally consistent with our previous findings [21,22,23,26]. In order to avoid secondary sound trauma to the animals’ ears, the highest intensity of the tone bursts used for all frequencies was no more than 90 dB SPL during the post-exposure ABR threshold tests in the current study. Therefore, the maximum 90 dB SPL post-exposure ABR threshold measured might not represent their actual hearing thresholds following acoustic trauma. Nevertheless, the degree of hearing loss at 16 kHz, 20 kHz, and 32 kHz was significantly greater than that at 8 kHz. This is thought to be the consequence of non-linear basilar mechanics, resulting in a larger amplitude of basilar membrane motion in response to the acoustic trauma frequency [27]. ABR thresholds were measured again after the behavioural confirmation of tinnitus, which was at 3 months following exposure to acoustic trauma. It was unexpected to see that the ABR thresholds completely recovered in the exposed ears of acoustic trauma animals in the aged group but not in the young group, as ABR thresholds have been shown to completely recover at 5–6 months following acoustic trauma in our previous studies, e.g., [21,22,23]. This discrepancy cannot be simply attributed to the age difference, since the age of the animals in the young group, at the time of acoustic trauma, was very similar to those animals used in our previous studies, e.g., [21,22,23]. A close inspection of the data revealed that, among the ten acoustic trauma rats in the young group, ABR thresholds remained elevated in three tinnitus-positive rats, while they returned to a normal level in four tinnitus-positive rats and three tinnitus-negative rats. This suggests that there might be some individual differences in the speed of recovery following acoustic trauma. It is also noteworthy that the last ABR thresholds were measured at 3 months after acoustic trauma in the present study, while they were measured at 5–6 months after acoustic trauma in previous studies, which may account for the incomplete recovery in the present study. Nevertheless, the elevated ABR thresholds in the exposed ear of the 3 rats should not have affected the tinnitus behavioural testing, as hearing in the unexposed ear was intact. The fact that both hearing-loss and hearing-intact animals exhibited behavioural evidence of tinnitus further supports the view that gross changes in ABR thresholds may not always accompany tinnitus, and a “hidden hearing loss” could be present with a normal audiogram [28].

In the present study, the tinnitus induction rate was 70%, which is inconsistent with the tinnitus induction rate of 30% to 80% reported in previous studies, e.g., [21,22,23]. In addition, the tinnitus perceived by these rats had acoustic features resembling 20 kHz and 32 kHz tones, which is in agreement with previous findings, e.g., [21,22,23]. Furthermore, an unexpected significant age effect was observed during tinnitus assessment, i.e., the young rats showed greater lick suppression than the aged rats following acoustic trauma. Given that the tinnitus induction rate was the same in both groups, the difference in the degree of lick suppression cannot be explained by age-related differences in tinnitus development. One possibility for the greater lick suppression in the young tinnitus animals could be that they experienced louder tinnitus than the aged animals, or they had the co-existence of hyperacusis, which might have made the tinnitus-resembling tones easier to be heard. Although hearing loss in the young animals in the present study was more persistent than that in the aged animals, there is no evidence to support a direct link between hearing loss, tinnitus loudness, and hyperacusis, and further investigations are needed to better understand the underlying mechanisms [29].

Increases in neuronal activity have been reported in the CN and the AC after acoustic trauma [4,30,31]. It has been suggested that the hyperactivity might be due to an imbalance between excitatory and inhibitory neurotransmissions [30,31,32]. Since extracellular levels of neurochemicals are believed to more closely reflect neurotransmissions and/or neurochemical function than the neurochemical levels in tissue homogenates [33,34], in vivo microdialysis is regarded as one of the best methods to monitor changes in neurotransmission. However, only two studies have investigated the relationship between the extracellular level of neurochemicals and tinnitus development. The first one, by Liu and colleagues [35], assessed serotonin release in the inferior colliculus (IC) and the AC of rats after salicylate administration using microdialysis. They found that serotonin levels increased to 268% of the baseline in the IC and 277% of the baseline in the AC at 2 h and 3 h after salicylate injection, respectively. The second study investigated time-dependent changes in amino acids in the IC of rats following acoustic trauma and tinnitus confirmation using microdialysis [21], which reported an increase in GABA levels a few hours and at 1 week after acoustic trauma. However, no changes in the basal levels of amino acids were found in tinnitus-positive animals 5 months later. The present study has the advantage of implanting two microdialysis probes in the CN and the AC, respectively, in the same rat, which made it possible to directly compare the extracellular levels of amino acids in the CN and the AC in relation to chronic tinnitus induced by acoustic trauma. We found no significant differences in any of the amino acids examined between sham and tinnitus-positive rats in either the CN or the AC during the baseline period, which is consistent with the findings of the IC using the same methods [21]. The lack of difference between the sham and tinnitus-positive animals was also reflected in the animo acids’ response to sound stimulation, where none of the amino acids examined responded differently between the two groups in either the CN or the AC.

Interestingly, serine levels were found to be lower in the aged animals in the AC in both the sham and tinnitus-positive groups, while the lowered level of threonine in the aged animals was only observed in the sham group. A general age-related decrease in amino acid levels, including serine and threonine, in different brain regions has been reported by comparing two groups of rats with a 26-month difference in age [36]. It is surprising to see that an age difference also existed in our current study, where there was only a 2-month difference between the young and aged groups. In the case of threonine, this age-related difference was only observed in the sham group. Since there was no difference in the ABR thresholds between the two age groups in the sham animals, the present results cannot be simply explained by age-related hearing loss [37]. In addition to the age-related difference, threonine and glycine levels in the CN varied in response to different frequencies of sound stimulation, and, in particular, the response was higher at the 4 kHz tone than the 16 kHz tone, which might be due to the variation in the placement of the microdialysis probes in different tonotopic regions of the CN.

Nevertheless, using microdialysis, the present study found that the presence of tinnitus did not affect the extracellular levels of amino acids, nor the amino acids’ response to sound stimulation, in either the CN or the AC in animals. Although the results do not support the view that tinnitus is generated by an imbalance between excitatory and inhibitory neurotransmissions [19,38,39,40,41], the findings are consistent with a microdialysis study of the IC [21], which reported no difference in amino acid levels between sham, tinnitus-negative, and tinnitus-positive animals. Further studies investigating a wide range of neurochemicals in both auditory and non-auditory brain regions are needed in order to understand tinnitus-related changes in neurotransmission.

## Data Availability

The raw data supporting the conclusions of this article will be made available by the authors on request.

## References

[1] Nondahl D.M., Cruickshanks K.J., Huang G.H., Klein B.E., Klein R., Javier Nieto F., Tweed T.S. (2011). Tinnitus and its risk factors in the Beaver Dam offspring study. Int. J. Audiol..

[2] Kaltenbach J.A. (2006). Summary of evidence pointing to a role of the dorsal cochlear nucleus in the etiology of tinnitus. Acta Otolaryngol. Suppl..

[3] Soussi T., Otto S.R. (1994). Effects of electrical brainstem stimulation on tinnitus. Acta Otolaryngol..

[4] Brozoski T.J., Bauer C.A., Caspary D.M. (2002). Elevated fusiform cell activity in the dorsal cochlear nucleus of chinchillas with psychophysical evidence of tinnitus. J. Neurosci..

[5] Zhang J.S., Kaltenbach J.A. (1998). Increases in spontaneous activity in the dorsal cochlear nucleus of the rat following exposure to high-intensity sound. Neurosci. Lett..

[6] Kaltenbach J.A., Afman C.E. (2000). Hyperactivity in the dorsal cochlear nucleus after intense sound exposure and its resemblance to tone-evoked activity: A physiological model for tinnitus. Hear. Res..

[7] Kaltenbach J.A., Zhang J., Finlayson P. (2005). Tinnitus as a plastic phenomenon and its possible neural underpinnings in the dorsal cochlear nucleus. Hear. Res..

[8] Brozoski T.J., Bauer C.A. (2005). The effect of dorsal cochlear nucleus ablation on tinnitus in rats. Hear. Res..

[9] Brozoski T.J., Wisner K.W., Sybert L.T., Bauer C.A. (2012). Bilateral dorsal cochlear nucleus lesions prevent acoustic-trauma induced tinnitus in an animal model. J. Assoc. Res. Otolaryngol..

[10] Eggermont J.J., Smith G.M. (1995). Synchrony between single-unit activity and local field potentials in relation to periodicity coding in primary auditory cortex. J. Neurophysiol..

[11] Norena A.J., Moffat G., Blanc J.L., Pezard L., Cazals Y. (2009). Neural changes in the auditory cortex of awake guinea pigs after two tinnitus inducers: Salicylate and acoustic trauma. Neuroscience.

[12] Engineer N.D., Riley J.R., Seale J.D., Vrana W.A., Shetake J.A., Sudanagunta S.P., Borland M.S., Kilgard M.P. (2011). Reversing pathological neural activity using targeted plasticity. Nature.

[13] Seki S., Eggermont J.J. (2003). Changes in spontaneous firing rate and neural synchrony in cat primary auditory cortex after localized tone-induced hearing loss. Hear. Res..

[14] Zhang J., Zhang Y., Zhang X. (2011). Auditory cortex electrical stimulation suppresses tinnitus in rats. J. Assoc. Res. Otolaryngol..

[15] De Ridder D., Ryu H., Møller A.R., Nowé V., Van de Heyning P., Verlooy J. (2004). Functional anatomy of the human cochlear nerve and its role in microvascular decompressions for tinnitus. Neurosurgery.

[16] Yang S., Weiner B.D., Zhang L.S., Cho S.J., Bao S. (2011). Homeostatic plasticity drives tinnitus perception in an animal model. Proc. Natl. Acad. Sci. USA.

[17] Petralia R.S., Al-Hallaq R.A., Wenthold R.J., Van Dongen A.M. (2009). Biology of the NMDA Receptor Frontiers in Neuroscience.

[18] Muly S.M., Gross J.S., Potashner S.J. (2004). Noise trauma alters D-[3H]aspartate release and AMPA binding in chinchilla cochlear nucleus. J. Neurosci. Res..

[19] Godfrey D.A., Kaltenbach J.A., Chen K., Ilyas O., Liu X., Licari F., Sacks J., McKnight D. (2012). Amino acid concentrations in the hamster central auditory system and long-term effects of intense tone exposure. J. Neurosci. Res..

[20] Godfrey D.A., Chen K., Godfrey M.A., Jin Y.M., Robinson K.T., Hair C. (2008). Effects of cochlear ablation on amino acid concentrations in the chinchilla posteroventral cochlear nucleus, as compared to rat. Neuroscience.

[21] Tan H.T., Smith P.F., Zheng Y. (2024). Time-dependent effects of acoustic trauma and tinnitus on extracellular levels of amino acids in the inferior colliculus of rats. Hear. Res..

[22] Zheng Y., Hamilton E., Begum S., Smith P.F., Darlington C.L. (2011). The effects of acoustic trauma that can cause tinnitus on spatial performance in rats. Neuroscience.

[23] Zheng Y., Vagal S., McNamara E., Darlington C.L., Smith P.F. (2012). A dose-response analysis of the effects of L-baclofen on chronic tinnitus caused by acoustic trauma in rats. Neuropharmacology.

[24] Paxinos G., Watson C. (2013). The Rat Brain in Stereotaxic Coordinates.

[25] Gurka M.J., Edwards L.J., Muller K.E. (2011). Avoiding bias in mixed model inference for fixed effects. Stat. Med..

[26] Zheng Y., Dixon S., McPherson K., MacPherson K., Smith P.F. (2015). Glutamic acid decarboxylase levels in the cochlear nucleus of rats with acoustic trauma-induced chronic tinnitus. Neurosci. Lett..

[27] Sellick P.M., Patuzzi R., Johnstone B.M. (1982). Measurement of basilar membrane motion in the guinea pig using the Mossbauer technique. J. Acoust. Soc. Am..

[28] Schaette R., McAlpine D. (2011). Tinnitus with a normal audiogram: Physiological evidence for hidden hearing loss and computational model. J. Neurosci..

[29] Eggermont J.J. (2021). Separate auditory pathways for the induction and maintenance of tinnitus and hyperacusis?. Prog. Brain Res..

[30] Baizer J.S., Manohar S., Paolone N.A., Weinstock N., Salvi R.J. (2012). Understanding tinnitus: The dorsal cochlear nucleus, organization and plasticity. Brain Res..

[31] Shore S.E., Wu C. (2019). Mechanisms of noise-induced tinnitus: Insights from cellular studies. Neuron.

[32] Sedley W., Parikh J., Edden R.A., Tait V., Blamire A., Griffiths T.D. (2015). Human auditory cortex neurochemistry reflects the presence and severity of tinnitus. J. Neurosci..

[33] Anderzhanova E., Wotjak C.T. (2013). Brain microdialysis and its applications in experimental neurochemistry. Cell Tissue Res..

[34] Plock N., Kloft C. (2005). Microdialysis–theoretical background and recent implementation in applied life-sciences. Eur. J. Pharm. Sci..

[35] Liu J., Li X., Wang L., Dong Y., Han H., Liu G. (2003). Effects of salicylate on serotoninergic activities in rat inferior colliculus and auditory cortex. Hear. Res..

[36] Banay-Schwartz M., Lajtha A., Palkovits M. (1989). Changes with aging in the levels of amino acids in rat CNS structural elements.3I. Glutamate and related amino acids. Neurochem. Res..

[37] Godfrey D.A., Chen K., O’Toole T.R., Mustapha A.I.A.A. (2017). Amino acid and acetylcholine chemistry in the central auditory system of young, middle-aged and old rats. Hear. Res..

[38] Dong S., Mulders W.H., Rodger J., Woo S., Robertson D. (2010). Acoustic trauma evokes hyperactivity and changes in gene expression in guinea-pig auditory brainstem. Eur. J. Neurosci..

[39] Barker M., Solinski H.J., Hashimoto H., Tagoe T., Pilati N., Hamann M. (2012). Acoustic overexposure increases the expression of VGLUT-2 mediated projections from the lateral vestibular nucleus to the dorsal cochlear nucleus. PLoS ONE.

[40] Browne C.J., Morley J.W., Parsons C.H. (2012). Tracking the expression of excitatory and inhibitory neurotransmission-related proteins and neuroplasticity markers after noise induced hearing loss. PLoS ONE.

[41] Dong S., Rodger J., Mulders W.H., Robertson D. (2010). Tonotopic changes in GABA receptor expression in guinea pig inferior colliculus after partial unilateral hearing loss. Brain Res..

